# Neuroscience Information Toolbox: An Open Source Toolbox for EEG–fMRI Multimodal Fusion Analysis

**DOI:** 10.3389/fninf.2018.00056

**Published:** 2018-08-24

**Authors:** Li Dong, Cheng Luo, Xiaobo Liu, Sisi Jiang, Fali Li, Hongshuo Feng, Jianfu Li, Diankun Gong, Dezhong Yao

**Affiliations:** ^1^The Clinical Hospital of Chengdu Brain Science Institute, MOE Key Lab for Neuroinformation, University of Electronic Science and Technology of China, Chengdu, China; ^2^School of Life Science and Technology, Center for Information in Medicine, University of Electronic Science and Technology of China, Chengdu, China

**Keywords:** EEG–fMRI, multimodal fusion, brain information, MATLAB toolbox, open source

## Abstract

Recently, scalp electroencephalography (EEG) and functional magnetic resonance imaging (fMRI) multimodal fusion has been pursued in an effort to study human brain function and dysfunction to obtain more comprehensive information on brain activity in which the spatial and temporal resolutions are both satisfactory. However, a more flexible and easy-to-use toolbox for EEG–fMRI multimodal fusion is still lacking. In this study, we therefore developed a freely available and open-source MATLAB graphical user interface toolbox, known as the Neuroscience Information Toolbox (NIT), for EEG–fMRI multimodal fusion analysis. The NIT consists of three modules: (1) the fMRI module, which has batch fMRI preprocessing, nuisance signal removal, bandpass filtering, and calculation of resting-state measures; (2) the EEG module, which includes artifact removal, extracting EEG features (event onset, power, and amplitude), and marking interesting events; and (3) the fusion module, in which fMRI-informed EEG analysis and EEG-informed fMRI analysis are included. The NIT was designed to provide a convenient and easy-to-use toolbox for researchers, especially for novice users. The NIT can be downloaded for free at http://www.neuro.uestc.edu.cn/NIT.html, and detailed information, including the introduction of NIT, user’s manual and example data sets, can also be observed on this website. We hope that the NIT is a promising toolbox for exploring brain information in various EEG and fMRI studies.

## Introduction

Since being first reported by Berger in 1929 (Berger, 1929), scalp electroencephalography (EEG) has been widely used to study brain function and dysfunction for approximately nine decades. Scalp EEG is a cost-effective and non-invasive technique that directly quantifies the electric fields of the brain at scalp sites with millisecond temporal resolution. However, due to the volume conduction effect (Yao et al., 2004) and potential closed electric fields of neural activity that are invisible to scalp recording (Nunez and Silberstein, 2000), EEGs have the limitations of poor spatial resolution (approximately one centimeter) and signal-to-noise ratio (SNR). In contrast, since it was developed in the 1990s, functional magnetic resonance imaging (fMRI) (Ogawa et al., 1990) has also been a famous non-invasive technique in basic cognitive neuroscience research (Logothetis, 2008). It measures changes in the blood oxygenation level-dependent (BOLD) signal, and voxel volumes with millimeter resolution are well suited to the anatomic scale of the hemodynamic changes in fMRI studies (Logothetis, 2008). Therefore, fMRI achieves millimeter spatial resolution, which results in superior functional localization power. However, fMRI BOLD signals do not reflect the neuronal activity directly; instead, they are a surrogate signal that contains a complex and slower transformation from neuronal activity to the BOLD signal, which is known as the hemodynamic response function (HRF) (Logothetis, 2008). Thus, fMRI is an indirect measure technique that has a relatively higher spatial resolution than EEG, but with low temporal resolution.

In view of the remarkable complementarity between them, EEG–fMRI multimodal fusion has become a highly desirable multimodal approach, which includes both high spatial and temporal resolutions in various human brain function (Laufs, 2012; Jorge et al., 2014; Ahmad et al., 2016) and dysfunction (Gotman and Pittau, 2011; Murta et al., 2015; Vitali et al., 2015) studies. Currently, there are three popular approaches to EEG–fMRI fusion (Biessmann et al., 2011; Huster et al., 2012; Laufs, 2012; Lei et al., 2012): (1) fMRI-informed EEG analysis, where EEG electromagnetic source reconstruction benefits from the spatial information of fMRI signals; in this approach, the ill-posed problem of EEG source imaging can be moderated with fMRI spatial constraints (Babiloni et al., 2005; Lei et al., 2011, 2015); (2) EEG-informed fMRI analysis, where EEG features in a specific frequency or time domain, such as epileptic discharge event onsets (Gotman and Pittau, 2011; Murta et al., 2014), event-related potential (ERP) amplitudes (Debener et al., 2006) and the power within specific EEG frequency bands (Moosmann et al., 2003; Mantini et al., 2007) are used to predict changes of fMRI BOLD signals; the basic assumption is that there is a linear and/or non-linear neurovascular coupling between simultaneous EEG and fMRI; (3) symmetric EEG–fMRI fusion, where EEG and fMRI data are analyzed jointly through a common generative model (Valdes-Sosa et al., 2009; Rosa et al., 2010) or in a common data space (Moosmann et al., 2008; Dong et al., 2015c) without possible bias.

However, the processing of EEG–fMRI multimodal fusion is a complicated task: it involves preprocessing of fMRI raw images, removing nuisance signals from fMRI data, re-referencing and marking interesting events (e.g., epileptic discharges) in simultaneous EEG data, extracting suitable EEG features in the time or frequency domain and convolving with different shapes of HRFs to generate predictive vectors, and analyzing EEG and fMRI data using a fusion method. This complexity not only constitutes a challenge to EEG–fMRI inexperienced researchers but also offers a large amount of flexibility in multimodal data analysis for experienced researchers. To successfully process EEG–fMRI multimodal data, a comprehensive and well-documented analysis toolbox is therefore required.

In this study, an open-source graphical user interface (GUI) toolbox, called Neuroscience Information Toolbox (NIT), is therefore developed for EEG–fMRI multimodal fusion. The NIT is based on some functions (e.g., functions to load EEG and fMRI data) in EEGLAB (Delorme and Makeig, 2004) and SPM (Ashburner, 2012) and runs on major computer operating systems (Windows 7/8/10 or Linux Ubuntu). It is designed to meet the needs of a wide variety of inexperienced and experienced researchers. To our knowledge, since the first version of NIT was released in 2015, at least 26 countries/areas and 795 visitors had already visited the NIT website^1^. The NIT can be downloaded for free at this website, and details of the NIT, including demos, example data and results, are also provided. The purpose of this technology report is to overview the general design, key features, illustrations and discussion of NIT.

## Materials and Methods

### Neuroscience Information Toolbox

#### NIT Programming Environment

Current NIT is developed in the MATLAB (version 2013a/2015a for 64 bit Win7/10 system) programming environment. MATLAB^2^ (The Mathworks, Inc., Natick, MA, United States) is a famous programming language that expresses matrix and array mathematics directly. It has many advantages, including compatibility with major operating systems (e.g., Windows/Linux), thousands of professional and reliable functions, a number of toolboxes for data analytics and statistics and the ability to scale analyses to run on clusters and GPUs. These make it easy-to-use for novice researchers to write small scripts/functions and use add-on toolboxes and for experienced researchers to develop new algorithms/toolboxes. Currently, MATLAB has become one of the most common programming environments for scientific research and toolbox development. Additionally, a number of neuroimaging toolboxes/packages, such as EEGLAB (Delorme and Makeig, 2004), FieldTrip (Oostenveld et al., 2011), SPM (Ashburner, 2012), GIFT^3^, EEG REST (Dong et al., 2017), and emiCCA toolbox (Dong et al., 2015c), have been developed based on MATLAB.

EEGLAB toolbox^4^ is a popular MATLAB toolbox for processing the EEG/MEG data, and it incorporates artifact removal, independent component analysis (ICA), time-frequency analysis, and event-related statistics (Delorme and Makeig, 2004). Moreover, EEGLAB extensions allow users to import various types of EEG data, which range from major EEG data formats (e.g., NeuroScan ^∗^.cnt data file) to specific/new data formats (e.g., NeuroScan Curry 6/7/8 data file). SPM^5^ is another popular MATLAB package that is designed for the analysis of EEG/fMRI/MEG/PET/SPECT data sequences, and it has become one of the most common MRI software packages in neuroscience (Ashburner, 2012). In our work, NIT relies heavily on extracted functions of EEGLAB (version eeglab14_1_0b) for (1) loading EEG data from all major EEG data formats; (2) processing EEG data, which includes filtering, re-referencing, time-frequency, and ICA analyses; (3) saving EEG data as EEGLAB data structures and conventions. Additionally, it relies on SPM12 (v6906) functions for (1) importing/saving MRI images and (2) fMRI preprocessing.

**FIGURE 1:**
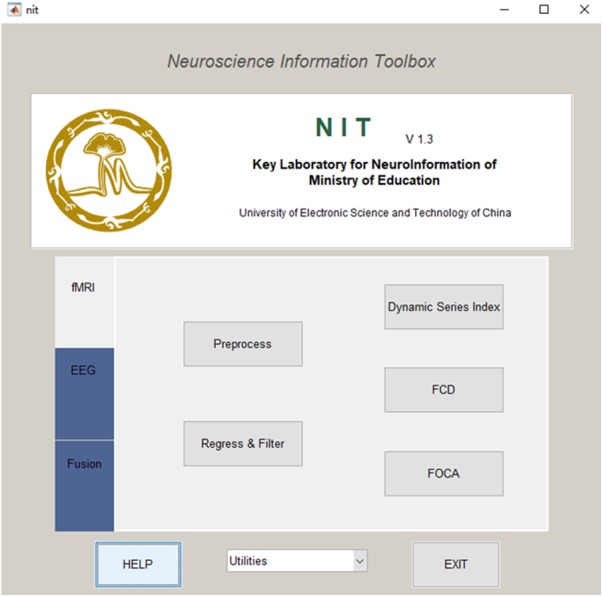
Main interface of NIT.

The installation of NIT is quite easy: (1) download the current release from http://www.neuro.uestc.edu.cn/NIT.html, unzip and add the path into MATLAB; (2) enter ‘nit’ as a command into the MATLAB command window, and the main interface of NIT will be shown (**Figure 1**).

#### General Design of NIT

**FIGURE 2:**
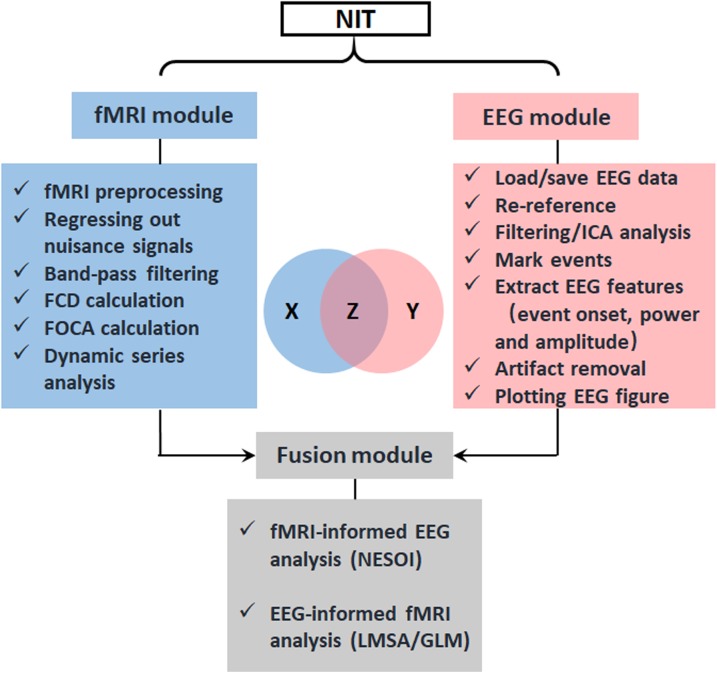
Design of NIT. NIT is designed as a convenient GUI, and it consists of fMRI, EEG and fusion modules. Conceptually, the EEG–fMRI multimodal fusion can be represented as information theoretic quantities, which are displayed as areas in a Venn diagram. X is corresponding to fMRI BOLD recordings, Y is corresponding to EEG electrophysiological signals, and Z is common information between EEG and fMRI revealed by fusion methods.

NIT is designed as a convenient GUI, which is an important aspect of an EEG–fMRI fusion analysis package. This approach can reduce the time required for both novice and experienced researchers to use NIT. As shown in **Figures 1, 2**, NIT contains three modules: (1) fMRI module, in which fMRI preprocessing, nuisance signal removing, bandpass filtering and calculation of resting-state measures are contained; (2) EEG module, in which artifact removal, the extraction of EEG features, re-referencing and marking interesting events, etc., are contained; and (3) fusion module, in which fMRI-informed EEG analysis and EEG-informed fMRI analysis are included. EEG and fMRI modules are used to generate temporal and/or spatial information for EEG–fMRI multimodal fusion analysis in the fusion module. Here, we will introduce the functions of NIT modules in detail.

In the EEG module, EEG data and the associated information from a single subject are stored in a structure array ‘EEG’ in the MATLAB workspace (the same as in EEGLAB data structures), while in most commercial systems it would correspond to an EEG file. The EEG data can be saved as an EEGLAB ^∗^.set file in the EEG module. These make it more convenient and easy to use for researchers who use NIT, especially for EEGLAB experienced users. In the fMRI module, NIfTI files are suggested, which contain affine coordinate definitions, repetition time and information on the left/right hemisphere. In fMRI preprocessing, if the converting option of the DICOM files is checked, NIT will convert the files to NIfTI images by calling dcm2nii in MRIcroN software^6^ on a Win7/10 system. Major EEG and fMRI data formats that are supported by NIT are shown in **Table 1**.

**Table 1:** File formats supported by NIT now.

Class of data	Manufacturer/file format
MRI formats	DICOM
	3D/4D Analyze images (^∗^.img)
	3D/4D NIfTI images (^∗^.nii or ^∗^.img)
EEG file formats	ASCII/Float file (^∗^.txt)
	MATLAB (^∗^.mat)
	EEGLAB (^∗^.set)
	Curry 6/7 (^∗^.dat/^∗^.dap/^∗^.rs3)
	BrainProducts/Brain Vision (^∗^.vhdr/.^∗^vmrk/^∗^.dat)
	NeuroScan (^∗^.cnt/^∗^.eeg)
	Biosemi/European Data Format (^∗^.bdf/^∗^.edf)
	BIOSIG (^∗^.edf/^∗^.edf+/^∗^.gdf/^∗^.bdf)
	BCI2000 (^∗^.bci2000)


### Key Features of NIT

#### Features of fMRI Module

##### fMRI preprocessing

**FIGURE 3:**
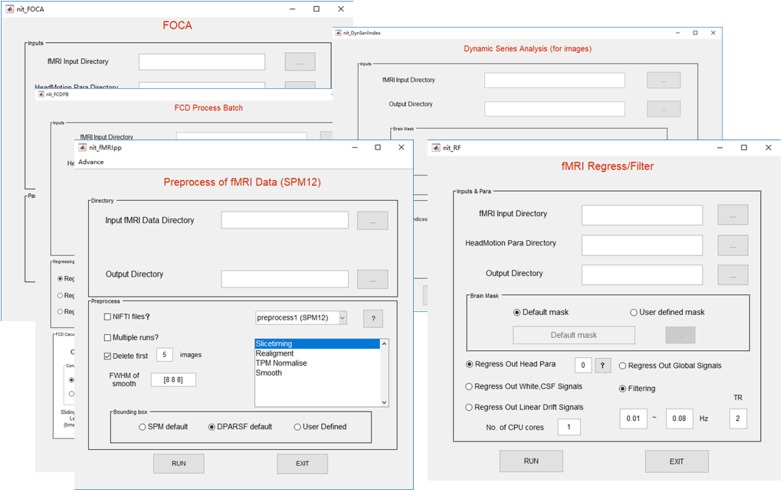
Interfaces of the fMRI module functions.

**FIGURE 4:**
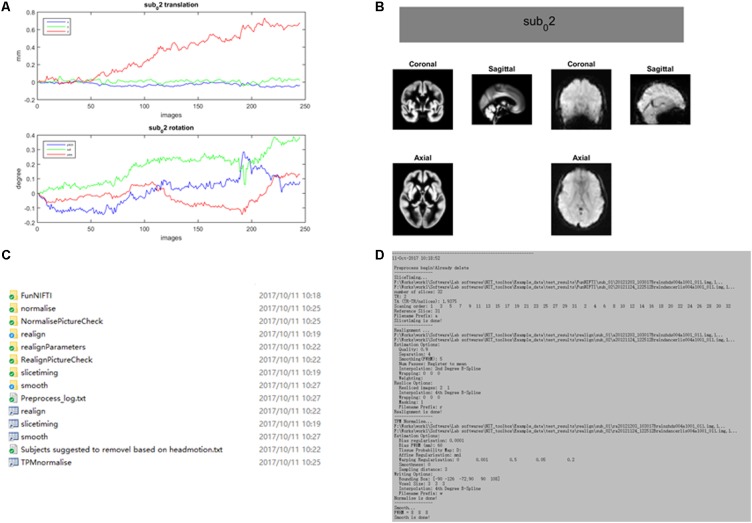
An example of fMRI preprocessing outputs using the NIT. All of the outputs in the NIT will be well organized and saved into separate folders. **(A)** Figure of head motion parameters; **(B)** TPM template and normalized image (first volume); **(C)** output folders; and **(D)** preprocessing log.

The preprocessing procedure comprises slice time correction, realignment, spatial normalization and smoothing in a conventional manner to process resting-state/task fMRI data. In SPM, the users must set SPM batches by hand for preprocessing the fMRI data of subjects, one by one. This approach could be inconvenient and cause a potential risk while processing hundreds of subjects. In NIT, preprocessing of batch files with default parameters will be automatically performed to preprocess the fMRI data based on SPM12 functions (**Figure 3**). At the same time, images generated from each preprocessing step will be organized and saved in separate folders (**Figure 4**). Currently, there are six fMRI preprocessing procedures in NIT: (1) preprocessing 1 (SPM12) comprised slice time correction, realignment, spatial normalization [using tissue probability map (TPM) template to normalize] and spatial smoothing; (2) preprocessing 2 (SPM12) comprised slice time correction, realignment, spatial normalization (using deformation parameters from individual T1 segmentation) and spatial smoothing; (3) preprocessing 3 (SPM12) comprised realignment, spatial normalization (using TPM template to normalize) and spatial smoothing; (4) preprocessing 4 (SPM8) comprised slice time correction, realignment, spatial normalization [using echo-planar imaging (EPI) template to normalize] and spatial smoothing; and (5) preprocessing 5 (SPM8) comprised realignment, spatial normalization (using EPI template to normalize) and spatial smoothing; and (6) smoothing based on SPM12.

##### Regressing out nuisance signals and filtering

In resting-state fMRI studies (e.g., functional connectivity analysis), there are two conventional procedures to remove possible nuisance signals (Biswal et al., 1995; Fox et al., 2005; Biswal et al., 2010; Dong et al., 2016c): (1) signals, including head motion parameters, linear trend, global signal (Fox et al., 2009; Weissenbacher et al., 2009), individual mean white and cerebrospinal fluid (CSF) signals, are always removed from fMRI data; and (2) temporal passband filtering (0.01–0.08 Hz) is conducted on fMRI data to reduce the effect of very low frequency and high frequency physiological noise. In the NIT, a linear regression model is utilized to remove these nuisance signals, and ideal bandpass filtering is realized. In addition, four sets of head motion regressors are provided, including 6-motion-parameter (R = [X, Y, Z, pitch, yaw, roll]), 12-motion-parameter (R and its derivative) (Power et al., 2014), and 24-motion-parameter (R, square of R, delay of R and its square, [R, R^2^, R*_t_*_-1_, R^2^*_t_*_-1_], where *t* and *t*-1 are the current and preceding time point) (Friston et al., 1996) and 36-motion-related parameters ([R, R^2^, R*_t_*_-1_, R^2^*_t_*_-1_, R*_t_*_-2_, R^2^*_t_*_-2_]) (Power et al., 2014).

##### Functional connectivity density

The high cognitive performance of humans can be supported by brain networks with energy-efficient hubs. Two novel measures, called local/global functional connectivity density (lFCD/gFCD), have been proposed to characterize the distributions of hubs in the brain (Tomasi and Volkow, 2010, 2011). The FCD measures have been used in various studies such as aging (Tomasi and Volkow, 2012), functional plasticity (Luo et al., 2014) and schizophrenia (Chen et al., 2017b). The lFCD of a given voxel is defined as the total number of functional connections between the voxel and its local cluster (correlation coefficient > a threshold and spatial adjacent voxels). The gFCD of a given voxel is the sum number of functional connections (correlation coefficient > a threshold) between the voxel and all brain voxels. At the same time, a long-range FCD (lrFCD), which is defined as gFCD minus lFCD, will be calculated in the NIT. To reduce the variability across subjects, the lFCD/gFCD/lrFCD will be divided by the global mean lFCD/gFCD/lrFCD value within the whole-brain mask. At the same time, dynamic FCD measures can be computed based on the sliding-window in the NIT. In addition, a vector of correlation thresholds (e.g., [0.2, 0.3, 0.4, 0.5, 0.6, 0.7, 0.8]) can be filled to calculate a range of FCD measures simultaneously, and the FCD calculation can be accelerated using parallel computing.

##### Spatiotemporal consistency of local neural activities

Recently, a new fMRI measure, called Four-dimensional (spatio-temporal) Consistency of local neural Activities (FOCA) (Dong et al., 2015a), has been proposed to characterize the local functional consistency of the brain by integrating temporal and spatial information in a local region. The FOCA measure has several advantages. (1) The FOCA measure can be a time-frequency domain method (Azeez and Biswal, 2017), and it is flexible in characterizing the local spontaneous activity. It emphasizes both the temporal homogeneity of local adjacent voxels and the regional stability of brain activity states (it can reflect local functional states) between neighboring time points. (2) It is a data-driven method without prior knowledge and practical parameter settings. (3) It has good reproducibility and reliability. The larger the FOCA value (value from 0 to 1) is, the higher the spatio-temporal consistency. The FOCA measure has been utilized to study schizophrenia (Chen et al., 2017a) and epilepsy (Dong et al., 2016a; Ma et al., 2017). It must be noted that temporal filtering and spatial smoothing have an impact on the spatial and temporal correlations in a FOCA calculation, respectively. One strategy is to remove these two steps from the preprocessing procedure. The FOCA measures will be divided by the global mean FOCA value within the whole-brain mask. The FOCA calculation can be accelerated using parallel computing in the NIT. More details of FOCA can be seen in the appendix in the NIT manual and the related paper (Dong et al., 2015a).

##### Dynamic series analysis

There is an increasing number of studies (Hutchison et al., 2013; Tomasi et al., 2016) on the temporal variability of the functional connectivity metrics to reveal the dynamic properties of the brain’s topology (Allen et al., 2014). In NIT, a range of basic indices is provided to assess the temporal variability of a series of functional measures/images, including the mean of a series, standard deviation of a series, coefficient of variation (standard deviation divided by the mean of a series), mean of point-by-point changes (1 - $\frac{v_{t}}{v_{t-1}}$, where *v_t_* and *v_t_*_-1_ are the values of the current and preceding time point) and mean of the relative ratio ($\frac{v_{t}}{v_{t-1}}$).

#### Features of the EEG Module

**FIGURE 5:**
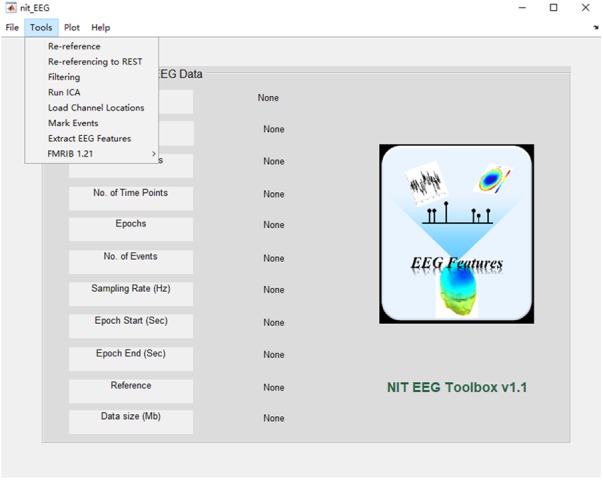
Main interface of the EEG module. The popup interface will display the primary information of the loaded EEG data, including the file name, set name, number of channels, time points, epochs and events, sampling rate, epoch start time, epoch end time, reference and data size.

**FIGURE 6:**
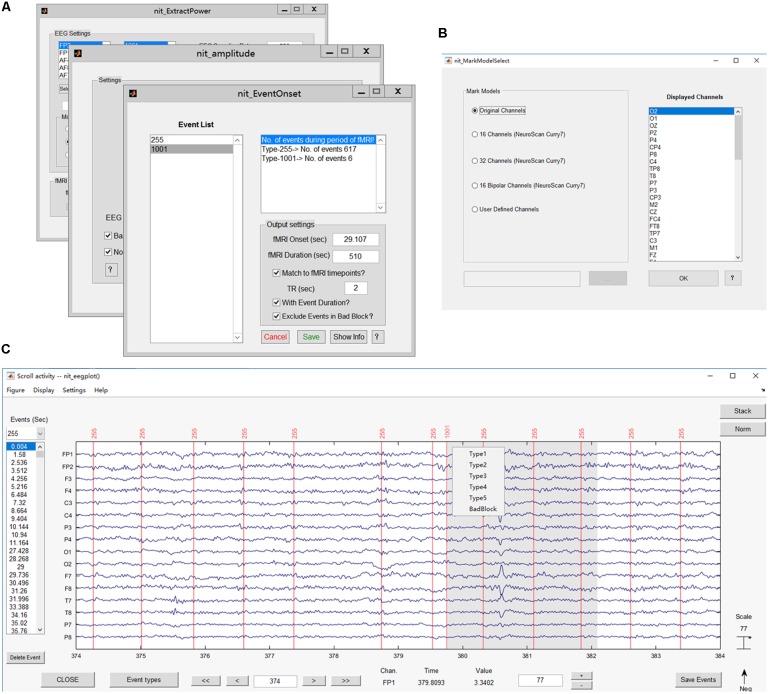
Main functions of the EEG module. **(A)** Interfaces for extracting EEG features, including onsets, power values and ERP amplitudes; **(B)** interface for selecting the channels to show; **(C)** plotting the EEG figure and marking interesting events (e.g., added label ‘1001’).

The NIT EEG toolbox, as an EEG module in the NIT, is mainly used to extract various features/measures (i.e., EEG information with a high temporal resolution) from simultaneous EEG data (**Figures 5,6A**). Additionally, fMRI regressors can be directly generated for EEG-informed fMRI analysis. Major EEG data formats supported by the NIT EEG toolbox can be seen in **Table 1**. The main functions available in the EEG module are as follows:

##### Re-reference

A number of references, comprised the neck ring (Katznelson, 1981), the tip of the nose (Andrew and Pfurtscheller, 1996), the vertex (Lehmann et al., 1998), unimastoid or ear (Başar et al., 1998), linked mastoids or ears (Gevins and Smith, 2000), average reference (Offner, 1950), and zero reference (named reference electrode standardization technique, REST) (Yao, 2001; Yao et al., 2005), have been proposed and used by different EEG researchers around the world. It is frequently useful to change the EEG reference offline. The NIT can convert an EEG dataset to an average, REST or to a new reference channel (or mean of several channels) offline by integrating the EEGLAB functions (Delorme and Makeig, 2004) and REST toolbox (Dong et al., 2017).

##### Filtering

Filtering continuous EEG data (e.g., bandpass or notch filtering) is a conventional frequency-domain procedure in EEG data analysis to remove artifacts (e.g., linear trends, power frequency and artificial peaks). Linear finite impulse response (FIR) filtering is utilized in the NIT by means of the MATLAB signal processing toolbox (routine ‘filtfilt’). If the signal processing toolbox is not present in MATLAB, then a fast Fourier transform (FFT) linear filter with the inverse Fourier transform will be used. Users can apply a low-pass, high-pass, or bandpass filter in NIT.

##### Independent component analysis

Independent component analysis was first used in EEG analysis in 1996 (Makeig et al., 1996a,b), and now, it is a common method that is used to decompose EEG epochs to remove potential artifacts or extract meaningful event-related components. NIT can perform ICA decomposition of input EEG data (epochs of an event) using the logistic infomax ICA algorithm (Bell and Sejnowski, 1995) with principal component analysis (PCA) dimension reduction. Noting that, in general, the number of ICs, N, can be set as the number of channels, while satisfying more than kN^2^ (k is a multiplier that increases as the number of channels increases) data sample points. An alternative good option to finding fewer stable components is using the PCA dimension reduction (set the number of PCs to retain) for insufficient data.

##### Load channel locations

While the EEGLAB functions of loading the channel locations in an EEG dataset are integrated into NIT, users can load or edit channel location information to plot EEG scalp topography or estimate EEG sources.

##### Mark events

Marking interesting events or bad blocks in continuous EEG data by hand is desired in EEG analysis, especially in finding and labeling epileptic discharges (Gotman and Pittau, 2011; Dong et al., 2015b, 2016b). Users can re-reference the EEG data in NIT and then show EEG figures by selecting a type of display model and marking interesting events (**Figures 6B,C**). There are five types of display models, including showing all channels, 16/32 channels based on the NeuroScan Curry7 system, 16 bipolar channels based on the NeuroScan Curry system and user-defined channels. In addition, the users can use the ‘Delete Event’ button to delete events one by one and press the ‘Save Event’ button to save events in a structure array ‘EEG’ in the MATLAB workspace.

##### Extract EEG features

In EEG-informed fMRI analysis, several specific EEG features over the time course of the EEG data are suitable, such as epileptic discharge event onsets (Gotman and Pittau, 2011; Murta et al., 2014), ERP amplitudes (Debener et al., 2006) and the power within specific EEG frequency bands (Moosmann et al., 2003; Mantini et al., 2007). Here, NIT can extract the EEG features, excluding the features in bad blocks and including event onsets, power values (time-frequency analysis) and ERP amplitudes, automatically match EEG feature variables to the fMRI time scale, and generate fMRI regressors that can be used for EEG-informed fMRI analysis. Note that the power value is calculated by $Y_{\mathrm{power}}\;=\;2*\frac{||Y|{|}^{2}}{\mathrm{length}\;\mathrm{of}\;\mathrm{epoch}\;\mathrm{signal}}$, where *Y* is a complex number calculated by the Fast-Fourier Transform (FFT), ||⋅|| represents complex modulus operations (using the ‘abs’ function of MATLAB), and extracting ERP amplitudes requires running ICA first to obtain stable components. More details can be seen in the NIT manual.

##### Artifact removal

Removals of the gradient and ballistocardiogram artifacts from raw EEG data are very important for EEG and fMRI fusion. The NIT can remove the fMRI-related artifacts by integrating an artifact removal plugin, which is a set of MATLAB tools developed by the University of Oxford Centre for Functional MRI of the Brain (FMRIB^7^).

##### Plotting EEG figures

In NIT, users can plot EEG figures with different display settings (e.g., number of channels to display, time range, channel labels and event on/off) and save an EEG image as a.JPEG/.TIFF/.BMP/.EPS/.PNG file (24 bit). The vertical axis is in the negative *y*-direction (↑Neg).

#### Features of the Fusion Module

##### Network-based source imaging

**FIGURE 7:**
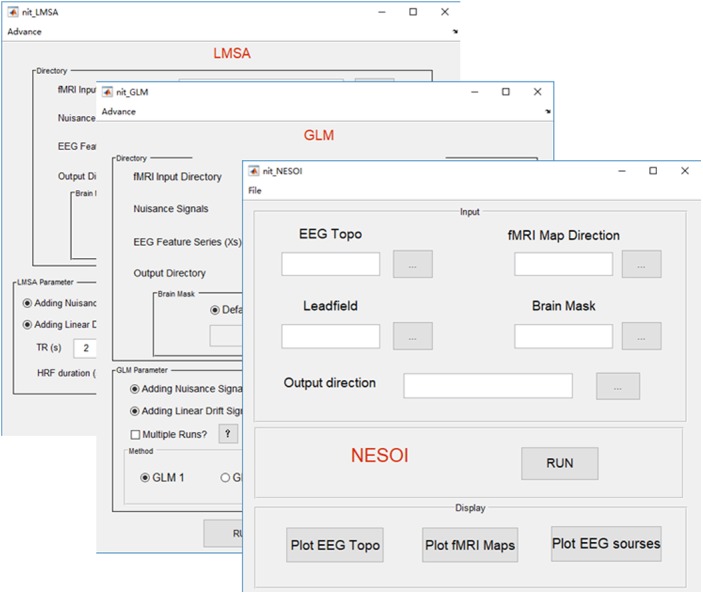
Main functions of the fusion module. There are three fusion methods in NIT, including NESOI, GLM, and LMSA.

Due to the high spatial resolution of fMRI, the functional networks during resting-state (Biswal et al., 1995, 2010) or a cognitive task (Mantini et al., 2009) could represent inter-regional correlations in neuronal variability, and such networks could facilitate EEG source estimation by moderating the ill-posedness of the original inverse problem (Huster et al., 2012; Lei et al., 2015). A new method, termed NEtwork based SOurce Imaging (NESOI), has been proposed to estimate the EEG sources (Lei et al., 2011). The novelty of NESOI is the utilization of multiple functional networks (obtained by fMRI with ICA) as spatial priors of EEG source estimation using parametric empirical Bayesian (PEB). NESOI is a useful approach to obtaining realistic EEG sources by combining the high temporal resolution EEG and high spatial resolution networks derived from fMRI. In NIT (**Figure 7**), NESOI is integrated with the default sources (6,144 dipoles), which are defined over a geometrically triangular grid based on the standard brain. More details about NESOI can be seen in a relevant paper (Lei et al., 2011) in the information provided in the NIT manual.

##### General linear model

Temporal integration of EEG–fMRI typically utilizes EEG features in the time or frequency domain (e.g., onsets, powers or ERP amplitudes) to inform the statistical mapping of fMRI, i.e., EEG-informed fMRI analysis (Gotman and Pittau, 2011; Huster et al., 2012; Murta et al., 2015). These EEG features are typically convolved with a canonical HRF or a set of HRFs with different shapes, and then, they are used to predict fMRI BOLD changes at the voxel-level using a general linear model (GLM) to obtain the statistical fMRI activation/deactivation maps related to the electromagnetic temporal signatures. In NIT, there are two types of GLM to estimate the BOLD changes: (1) GLM1, which is a conventional way that all regressors are estimated in a linear model voxel by voxel. It is suitable for event or block design fMRI analysis; and (2) GLM2, which is designed to investigate the EEG discharge related BOLD changes in epilepsy (Bagshaw et al., 2004). For each fMRI voxel, the fMRI data are analyzed several times (e.g., once for each of the different HRF peaks, at 3, 5, 7, and 9 s), and the maximal *T*-value is chosen to account for the activation of this voxel. In addition, NIT provides different HRF shapes, including single gamma, standard SPM (Friston et al., 1998) and Glover (Glover, 1999) HRFs. Nuisance signals, such as head motion, linear trend, white and CSF signals, can also be added into the GLM. Note that before the GLM calculation, a high-pass filter is used to remove the low frequency fMRI noise (the cut-off period in seconds is 128 s), and global mean normalization is also conducted (i.e., data = 100⋅data/global mean). More details of the GLM in the NIT can be seen in the NIT manual.

##### Local multimodal serial analysis

In EEG-informed fMRI analysis in epilepsy, there are also some problems using simple GLM: one is the variation in the HRFs in patients; in other words, a canonical HRF might not be the best model for the BOLD changes related to spikes (Masterton et al., 2010). Another is the low SNR in the EEG (e.g., gradient and ballistocardiogram artifacts) and fMRI (due to the high spatial resolution) (Tabelow et al., 2009) data. Simple GLM might be difficult to uncover the weak fMRI BOLD changes that are related to the EEG features in epilepsy. Therefore, a new method, called local multimodal serial analysis (LMSA), has been proposed to compensate for these deficiencies in multimodal integration (Dong et al., 2015b). The novelty of LMSA is serially fusing EEG and fMRI in the local region to efficiently capture the potential brain functional activities. There are two key steps in LMSA: (1) for a given voxel *i*, canonical correlation analysis (CCA) is used to obtain the significant canonical variate (i.e., *v_i_*), which corresponds to the maximal correlation between the EEG feature set (i.e., the lagged function matrix of the EEG features) and the fMRI set of the local time series (27 adjacent voxels); and then, (2) a multiple linear regression model is used to estimate the activity of the voxel *i* that corresponds to *v_i_*. Finally, the abovementioned procedure is performed for all voxels, and the T-map of the estimated regression coefficients can be obtained. The NIT realizes the functions of LMSA with global mean normalization. The users can also use the LMSA module to show the estimated HRF of a voxel calculated by LMSA. More details about LMSA can be seen in the appendix of the NIT manual.

### Illustrations

To validate and illustrate the usage of the NIT toolbox, as an example, we performed fMRI preprocessing, FOCA, FCD and EEG-informed fMRI analyses (GLM2) using the simultaneous EEG–fMRI data of a previous paper (Dong et al., 2016b). Note that four more example datasets with results (including a resting-state fMRI example, NESOI example, EEG-informed fMRI analysis in epilepsy and EEG-informed fMRI analysis for EEG–fMRI data during a P300 visual task) are also provided at the NIT website (see footnote 1) to illustrate the usage of NIT. Users can use these datasets to walk through NIT while referring to the NIT manual (Chinese and English versions are available).

#### Participants

A total of 18 juvenile myoclonic epilepsy (JME) patients were gathered in this work (6 males/12 females, age range = 15–34 years, mean age = 21 years, standard deviation = 7 years). All patients were diagnosed by two neurologists according to clinical information consistent with the International League Against Epilepsy (ILAE) guidelines. Written consent forms in accordance with the Declaration of Helsinki were received from all patients. More details on the participants, such as the demographic information and clinical characteristics, can be found in the related article (Dong et al., 2016b). The experiment was approved by the local ethics committee of University of Electronic Science and Technology of China (UESTC).

#### Simultaneous EEG–fMRI Data Acquisition

The EEG data were recorded using a 64-channel (62 EEG electrodes according to 10-20 cap system, 1 electrooculogram and 1 electrocardiogram electrodes) MR compatible EEG system (NeuroScan, Charlotte, NC, United States). The sampling rate was set at 5 kHz.

The fMRI data were gathered using the 3-Tesla MRI scanner (Discovery MR750, GE, United States). A 3-dimensional fast spoiled gradient echo sequence was used to obtain T1 structural images (152 axial slices, TR = 5.936 ms, TE = 1.956 ms, flip angle = 9°, voxel size = 1 mm × 1 mm × 1 mm, field of view = 25.6 cm × 25.6 cm, thickness = 1 mm), and a gradient-echo echo-planar imaging sequence was used to obtain functional images (35 slices per volume, TR = 2 s, TE = 30 ms, flip angle = 90°, matrix size = 64 × 64, field of view = 24 cm × 24 cm, thickness = 4 mm). All of the patients were instructed to close their eyes and relax without falling asleep during the scanning. There are five repeated runs (510 s × 5 = 40.25 min) for each patient. Here, for each JME patient, one run with small head motion (rotation < 1°and translation < 1 mm) and high-quality of both the EEG and fMRI data (e.g., no eye blinks and no falling asleep) was used.

#### Data Analysis

For the EEGs, removing the gradient and ballistocardiogram artifacts in the raw EEG data, bandpass filtering (1–30 Hz) and down-sampling to 250 Hz were conducted preliminarily. Then, using NIT, all of the EEG data were re-referenced to the Cz reference and marked with the onsets of generalized spike-wave discharges (GSWDs) by two experienced neurologists (independently, both in agreement).

For fMRI, the functional images were analyzed in a conventional fashion using NIT. The fMRI data preprocessing comprised deleting the first five images, slice time correction, realignment, spatial normalization using the EPI template (3 mm × 3 mm × 3 mm, bounding box with the Data Processing Assistant for Resting-State fMRI (DPARSF) default [-90, -126, -72; 90, 90, 108]) and spatial smoothing (8-mm full-width at half maximum). The preprocessing analysis was conducted using ‘preprocess 4 (SPM8)’ in NIT. Then, normalized images were used to calculate the FOCA and FCD measures using NIT. Default settings of the FOCA calculation are regressing out six head motion parameters, white matter, CSF and linear trend signals, selecting point connection criterion (local 27 voxels) and setting TR = 2 s (Dong et al., 2015a). Default settings of the FCD calculation are regressing out six head motion parameters, white matter, CSF and linear trend signals, bandpass filtering (0.01–0.08 Hz), selecting static connectivity with the line connection criterion and setting the correlation threshold = 0.6 (Tomasi and Volkow, 2010, 2011).

For EEG-informed fMRI analysis, traditional GLM in epilepsy was conducted on smoothed fMRI images using NIT. Onsets of GSWDs generated from EEG were used to obtain fMRI regressors, which convolved with four SPM HRFs and peaked at 3, 5, 7, and 9 s (Bagshaw et al., 2004). Linear trend signal and six head motion parameters were added as covariates in GLM. The type of GLM2 was selected in NIT, and an *F*-test was used to assess the significant BOLD changes related to the EEG discharges in the JME patients.

## Results and Discussion

**FIGURE 8:**
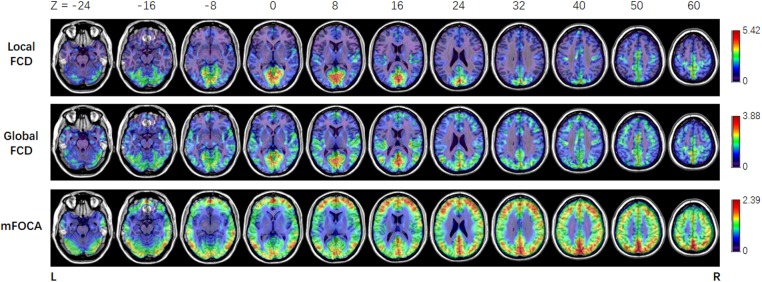
Results of mean normalized local FCD, global FCD and FOCA maps in JME patients.

**FIGURE 9:**
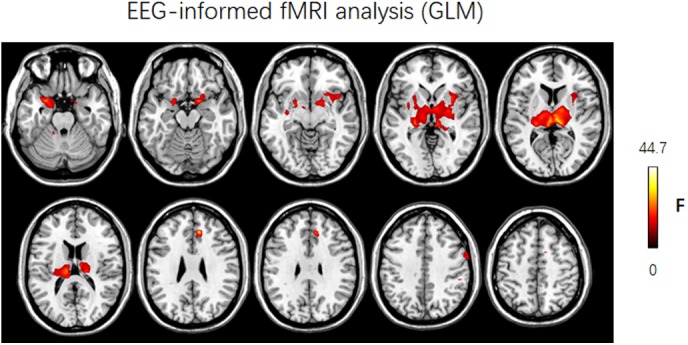
Results of discharge-related hemodynamic changes in JME patients revealed by traditional EEG-informed fMRI analysis (GLM2, *P* < 0.005, uncorrected). F, *F*-value; Left is left.

In this work, the simultaneous EEG–fMRI data of JME patients were used to illustrate the usage of NIT. **Figure 8** showed that the brain regions in which the FOCA values were greater than the mean FOCA values of the whole brain mainly included the bilateral cerebellum (Brodmann Area BA19), middle temporal lobes (BA21), precuneus (BA7), angular gyrus (BA39), frontal lobes (BA46/10/9) and visual cortex (BA17/18). The brain regions with higher local FCD values mainly contained the bilateral lingual gyrus (BA17/18), precuneus (BA7) and postcentral gyrus (BA4), while the regions with greater global FCD values included the bilateral lingual and calcarine gyrus (BA17/18), middle temporal lobes (BA21), superior temporal lobes (BA48), precuneus (BA7) and supplementary motor area (BA6). The current results of the resting-state fMRI measures are consistent with the previous FOCA (Dong et al., 2015a; Chen et al., 2017a; Ma et al., 2017) and FCD (Tomasi and Volkow, 2010, 2011, 2012; Luo et al., 2014; Chen et al., 2017b) studies. **Figure 9** demonstrated that changes in the BOLD signals related to GSWDs were mainly found in the right anterior cingulate (BA32), right precentral gyrus (BA44), bilateral thalamus, pallidum and putamen using traditional EEG-informed fMRI analysis. These results provided further evidence that a thalamofrontal network might be associated with the modulation and propagation of epileptic activity in JME, which were in line with a number of studies in idiopathic generalized epilepsy (Norden and Blumenfeld, 2002; Gotman et al., 2005; Li et al., 2009; Luo et al., 2012) and JME (Pulsipher et al., 2009; Koepp et al., 2014; Dong et al., 2016b; Jiang et al., 2018). The above-mentioned illustrations of multimodal analyses by using NIT validated its correctness and demonstrated its effectiveness.

The NIT should still be greatly improved in the future. For example, a potential development of NIT is connect it to the cloud, i.e., integrating it into neuroscience cloud computing platforms such as the Canadian brain imaging research platform (CBRAIN) (Sherif et al., 2014) and WeBrain^8^. Furthermore, more functions, such as basic statistical analysis and other EEG–fMRI fusion methods, will be integrated into NIT in the future. In addition, although there is a detailed NIT user’s manual contained in the NIT package, extensive files and information related to NIT is available at http://www.neuro.uestc.edu.cn/NIT.html. This website includes the latest version release of NIT, a brief introduction of EEG–fMRI fusion, example data sets and corresponding results for demos and a list of references. These make it more efficient and convenient for customers to enjoy NIT, even those who have little programming experience. Bugs are an inevitable part of any software development project. It is appreciated when users report bugs, constructive suggestions and/or problems about NIT via email to the authors (Lidong@uestc.edu.cn) or leave a message in the NIT community (see footnote 1).

## Conclusion

Based on MATLAB, NIT provides an easy-to-use, flexible and transparent package for EEG–fMRI multimodal fusion. NIT’s GUI, as well as support documents and detailed demos, dramatically reduce the time required for users to learn the usage of NIT. At the time of this writing, the website and system has been publicly available and improved for approximately 3 years, and it has become more stable and mature. We hope that this user-friendly NIT could make the relatively novel technique of multimodal fusion easier to study, especially for applications in various EEG and fMRI studies.

## Author Contributions

LD, CL, and DY conceived and designed the work. LD and JL wrote code. LD, SJ, and FL acquired the example data. LD, XL, HF, SJ, FL, JL, and DG analyzed the data and tested the software. LD, CL, DY, and DG wrote and revised the manuscript. All authors revised the work for important intellectual content. All of the authors have read and approved the manuscript.

## Conflict of Interest Statement

The authors declare that the research was conducted in the absence of any commercial or financial relationships that could be construed as a potential conflict of interest.

## Footnotes

1 http://www.neuro.uestc.edu.cn/NIT.html

2 https://www.mathworks.com/

3 http://mialab.mrn.org/software/gift/index.html

4 https://sccn.ucsd.edu/eeglab/index.php

5 http://www.fil.ion.ucl.ac.uk/spm/

6 http://people.cas.sc.edu/rorden/mricron/index.html

7 https://fsl.fmrib.ox.ac.uk/eeglab/fmribplugin/

8 http://webrain.uestc.edu.cn/
